# Supplementary material for: Electrocatalytic Degradation of Levofloxacin, a Typical Antibiotic in Hospital Wastewater
Source: Materials (Basel). 2021 Nov 11;14(22):6814. doi: 10.3390/ma14226814 (PMC8621070; doi:10.3390/ma14226814)
Supplement: Supplementary file 1 [file materials-14-06814-s001.zip › materials-1386639-supplementary.pdf]

materials

MDPI

Electrocatalytic Degradation of Levofloxacin, a Typical Antibiotic in Hospital Wastewater

Hongxia Lv [1,†], Peiwei Han [2,†], Xiaogang Li [1], Zhao Mu [3], Yuan Zuo [1], Xu Wang [1], Yannan Tan [4], Guangxiang He [1], Haibo Jin [1], Chenglin Sun [4], Huangzhao Wei [4,*] and Lei Ma [1,*]

1 Beijing Key Laboratory of Fuels Cleaning and Advanced Catalytic Emission Reduction Technology, College of New Materials and Chemical Engineering, Beijing Institute of Petrochemical Technology, Beijing 102617, China; 2019520036@bipt.edu.cn (H.L.); micklxg@163.com (X.L.); 2018310072@bipt.edu.cn (Y.Z.); wxonly0120@163.com (X.W.); hgx@bipt.edu.cn (G.H.); jinhaibo@bipt.edu.cn (H.J.)
2 Guangzhou Institute of Energy Conversion, Chinese Academy of Sciences, Guangzhou 510640, China; hanpeiwei025@163.com
3 Institute of Applied Chemical Technology for Oilfield, College of New Materials and Chemical Engineering, Beijing Institute of Petrochemical Technology, Beijing 102617, China; muzhao@bipt.edu.cn
4 Dalian Institute of Chemical Physics, Chinese Academy of Sciences, Dalian 116023, China; yntan@dicp.ac.cn (Y.T.); clsun@dicp.ac.cn (C.S.)
* Correspondence: whzhdicpwtg@dicp.ac.cn (H.W.); malei@bipt.edu.cn (L.M.)
† Two authors contribute the same.

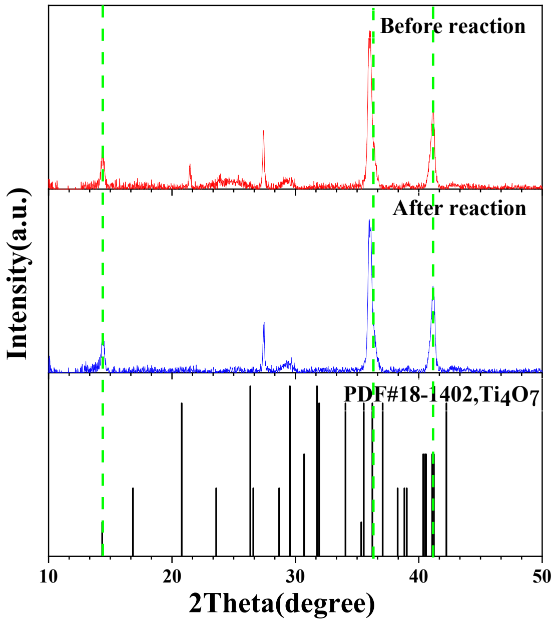

Figure S1. XRD spectra before and after electrooxidation reaction.

Table S1. The weight percentage and atomic percentage of each element on the surface of titanium suboxide before and after the reaction.

Titanium Suboxide Anodes	Weight Percentage		Atomic Percentage	
	O(%)	Ti(%)	O(%)	Ti(%)
Titanium oxide anode before reaction	46.35	53.65	72.12	27.88
Titanium oxide anode after reaction	43.50	56.50	69.74	30.26

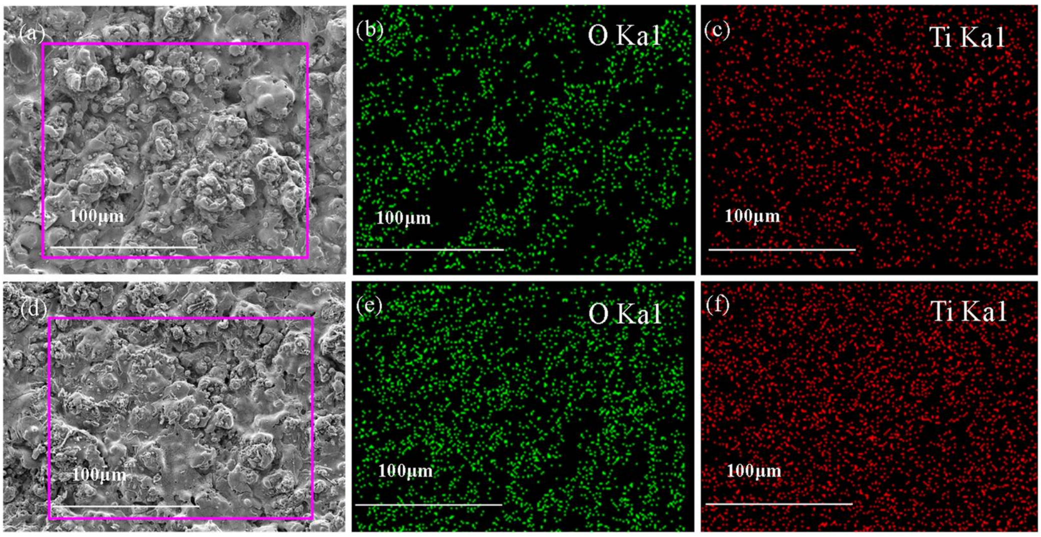

Figure S2. EDS spectrum of the titanium suboxide electrode before (**a**), (**b**), (**c**) and after (**d**), (**e**), (**f**) the electrooxidation reaction.

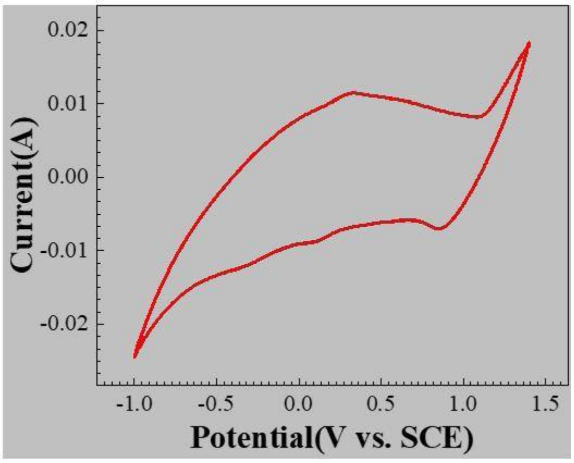

(**a**)

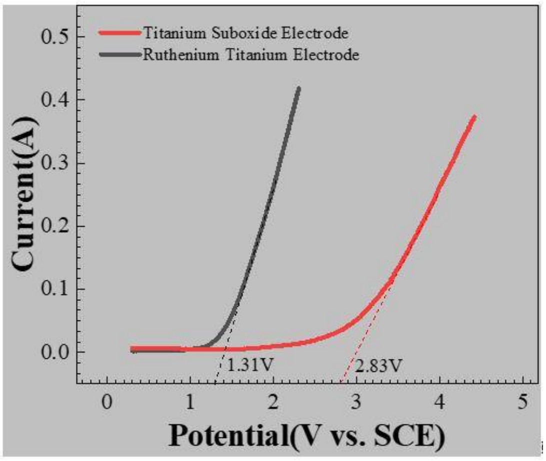

(**b**)

Figure S3. Titanium Suboxide Electrode (**a**) cyclic voltammetry (CV) curve of titanium suboxide electrode in 100ppm levofloxacin, 1mol/L Na_2SO_4 solution, scanning rate: 50mV/S (**b**) linear sweep voltammetry (LSV) curve of ruthenium titanium electrode and titanium suboxide electrode in 10 mmol/L potassium ferricyanide, 1mol/L Na_2SO_4 solution, scanning rate: 50mV/S.

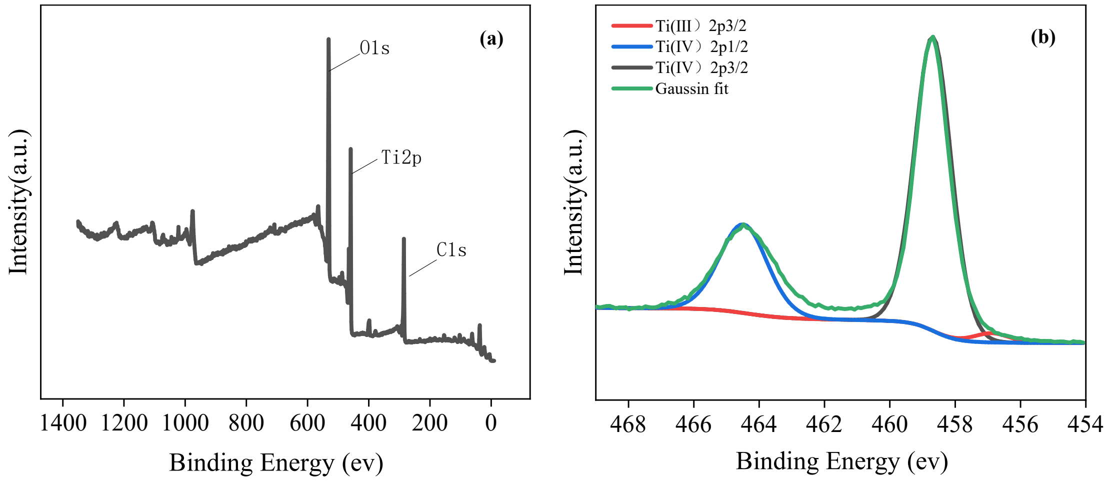

Figure S4. XPS spectrum of the titanium suboxide (**a**) full-scan spectrum (**b**) Ti 2p.
